# Optimisation of hypocrellin production in *Shiraia*-like fungi via genetic modification involving a transcription factor gene and a putative monooxygenase gene

**DOI:** 10.1080/21501203.2023.2295406

**Published:** 2023-12-25

**Authors:** Zi-Min Lu, Run-Tong Zhang, Xiao-Bo Huang, Xue-Ting Cao, Xiao-Ye Shen, Li Fan, Cheng-Lin Hou

**Affiliations:** College of Life Science, Capital Normal University, Beijing, China

**Keywords:** *Shiraia*-like fungi, hypocrellins, transcription factor, monooxygenase

## Abstract

*Shiraia*-like fungi, which are rare parasitic fungi found around bamboo, play an important role in traditional medicine. Their main active component, hypocrellin, is widely used in medicine, food, and cosmetics. By comparing strains with different hypocrellin yields, we identified a transcription factor (*SbTF*) in the hypocrellin biosynthesis pathway. *SbTF* from high-yielding zzz816 and low-yielding CNUCC C72 differed in its protein structure. Subsequently, *SbTF* from high-yielding zzz816 was overexpressed in several strains. This stabilised the yield in zzz816 and significantly increased the yield in low-yielding CNUCC C72. Comparing downstream non-essential genes between wild type and *SbTF*-overexpressing CNUCC C72 showed that *SbMNF* was significantly up-regulated. Therefore, it was selected for further study. *SbMNF* overexpression increased the hypocrellin yield in low-yielding CNUCC C72 and altered the composition of compounds in high-yielding CNUCC 1353PR and zzz816. This involved an increased elsinochrome C yield in CNUCC 1353PR and an increased hypocrellin B yield in zzz816 (by 2 and 70.3 times that in the corresponding wild type, respectively). This study is the first to alter hypocrellin synthesis to alter the levels of one bioactive agent compared to another. The results provide new insights regarding genetic modification and will help to optimise fungal fermentation.

## 1. Introduction

Various secondary metabolites (SMs) produced during the growth and development of fungi have been discovered. These bioactive components play a crucial role in resisting environmental stresses and cytotoxins, as well as facilitating cell communication (Alberti et al. 2017). Some fungal SMs are highly beneficial for humans. For example, penicillin is used for the prevention of surgical wound infections. Other fungal SMs are also used as antibiotics and other treatments, and so they have revolutionised the field of medicine, contributing to the treatment of various diseases (Aly et al. 2011). Additionally, various fungal SMs exhibit diverse biological activities that are beneficial in fields such as food, agriculture, and environmental protection.

Perylenequinones (PQs) from endophytic or pathogenic fungi of plants have attracted increasing attention as photosensitisers. These compounds belong to the polyketide family, which includes elsinochrome from *Elsinoe*, cercosporin from *Cercospora*, and phleichrome and hypericin from plants. Hypocrellins, the main active component of the traditional medicinal fungi known as *Shiraia*-like species, are also PQs. PQs are commonly used in traditional medicine to treat rheumatoid arthritis, gastric diseases, skin diseases, and various superficial microvascular diseases. Recent reports have also demonstrated their bacteriostatic, anti-inflammatory, and anti-viral properties. As natural photosensitisers, PQs produce reactive oxygen species when exposed to light, so they have great potential for use in photodynamic anti-tumour therapy (Tang et al. 2019). In particular, during fermentation culture, *Shiraia*-like species can synthesise various PQs, including hypocrellins (HA, HB, HC) and elsinochromes (EA, EB, EC), both of which are photosensitisers and exhibit good bioactivity against various factors that negatively affect the fungi (Morakotkarn et al. 2008; Liang et al. 2009; Yang et al. 2009; Tong et al. 2017).

Furthermore, structural modification of hypocrellin and hypocrellin derivatives is anticipated to improve their properties and extend their applications. First, Gao et al. (2012) developed hypocrellin-loaded gold nanocages and showed that they exhibited high two-photon efficiency for photothermal cancer therapy *in vitro*. Second, hypocrellin B (HB) was modified using 1,2-diamino-2-methyl propane to create DPAHB, which self-assembles into nanovesicles using polyethylene glycol (PEG)-poly(lactic-co-glycolic acid) (PLGA). These nanovesicles had high photothermal stability, enhanced accumulation in tumours, and a suitable biodegradation rate (Zheng et al. 2018). Third, Li et al. (2021) discovered that a new hypocrellin analogue designated hypomycin F, together with five known compounds, inhibited SAR-CoV-2 entry into cells. Lastly, ethylenediamine-modified elsinochromes, which can absorb light at longer wavelengths, were found to have higher bioactivity than non-modified elsinochromes (Qiao et al. 2012).

Due to the complex structures, excessive number of steps, low yields, and environmental pollution, chemically synthesising hypocrellins and their analogues is rarely done in practice (Mulrooey et al. 2012). The extraction of natural products from fruiting bodies produces limited results, and this process can disrupt the ecological balance (Tong et al. 2017; Liu et al. 2018). Therefore, fermentation is currently the preferred method for obtaining hypocrellins.

Over the past two decades, various processes have been used to improve hypocrellin production during submerged fermentation of *Shiraia*-like species. The hypocrellin yield has reached 90–8,632 mg/L (Liu et al. 2016; Lei et al. 2017; Deng et al. 2020). The addition of the surfactant TritonX-100, the growth factor GZUIFR-TT1, and hydrogen peroxide during the culture has effectively increased the hypocrellin yield (Yang et al. 2009; Cai et al. 2011; Du et al. 2013). Our laboratory also isolated the industrial strain zzz816, which exhibits higher production efficiency and relatively stable production. After mutagenesis via cobalt-60 γ-irradiation, the hypocrellin yield of zzz816 was further increased by 414.9%, but it was unstable (Liu et al. 2016). After broader screening for high-yielding strains with increased production stability, the more efficient wild type CNUCC 1353PR was identified (Tong et al. 2021). However, the increases in output as a result of these measures remain insufficient to meet the growing demand. Therefore, mechanistic analysis of hypocrellin biosynthesis and genetic modification are expected to play key roles in addressing the production bottlenecks (Chen et al. 2022).

The biosynthesis of PQs such as hypocrellins (and cercosporin and elsinochrome, which have similar structures) can be attributed to the polyketide synthesis pathway. Briefly, the three main enzymes involved in PQ biosynthesis are: polyketide synthase (PKS), which is responsible for the core PQ structure; phenol coupling enzymes, which catalyse the dimerisation of the core structure; and pruning enzymes, which modify functional groups to generate PQs with different structures (Obermaier et al. 2019).

The hypocrellin biosynthesis gene cluster was verified by comparing the transcriptome of the hypocrellin A (HA)-producing strain *Shiraia bambusicola* S4201-W and its UV-treated mutant S4201-D1 without HA production (Zhao et al. 2016). A single PKS is regarded as the key enzyme that forms the basic skeleton (Newman and Townsend 2016; Zhao et al. 2016; Deng et al. 2017; Gao et al. 2018; Li et al. 2019). A series of functional genes around it modify the intermediate product to produce hypocrellins. Genes encoding hydroxylase (hyd) and monooxygenase (mono) proteins have been shown to be involved in hypocrellin biosynthesis, and overexpression of the critical genes may increase production (Li et al. 2019). Zhao et al. (2020) discovered the zinc finger transcription factor Zftf through genome sequencing and analysis of the hypocrellin-producing strain *S. bambusicola* S4201.

In this study, for the first time, we genetically modified various *Shiraia*-like industrial strains by overexpressing functional genes, and thereby increased hypocrellin production. The special transcription factor *SbTF* and the downstream putative monooxygenase gene *SbMNF* (identified as a non-core gene around the hypocrellin biosynthesis gene cluster) represent potential breakthroughs for developing high-yielding strains. The function of the non-core gene *SbMNF* in the biosynthesis pathway provides a new perspective for strain transformation and hypocrellin production optimisation.

## 2. Materials and methods

### 2.1. Strains and plasmid

*Shiraia*-like species CNUCC 1353PR = CFCC 55715 and zzz816 were obtained from the China Forestry Culture Collection Center (CFCC). *Shiraia*-like species CNUCC C72 was obtained from the Capital Normal University Culture Collection Center (CNUCC). The following strains were used as the wild type recipients in the transformation experiments: (1) high-yielding strain CNUCC 1353PR, (2) high-yielding but unstable strain zzz816, (3) low-yielding strain CNUCC C72.

A binary vector was constructed based on the pAg1-H3 backbone (provided by Gang Liu, Institute of Microbiology, Chinese Academy of Sciences, Beijing, China), which harbours a hygromycin B resistance gene as a selection marker under the control of the fungal promoter *PdgA* and terminator *TtrpC*. An *SbTF* (transcription factor) fragment (1.3 kb) and an *SbMNF* (putative monooxygenase gene) fragment (1.3 kb), amplified from the *S. bambusicola* genome, were each separately incorporated into the plasmid. Linearised pAg1-H3 was ligated to each gene fragment using a ClonExpress^TM^ II One Step Cloning Kit (Vazyme Biotech, Nanjing, China). Both gene fragments were driven by the *PdgA* promoter. The primers used are listed in Table 1.

**Table 1.** Primers.

Primer	Sequence (5’–3’)
*SbTF*-F	ATGGCCACTCAACTCCCTAC
*SbTF-*R	CTATCCCGATCCGTTCAAAT
YZ*-SbTF*-F	CCCATCCCTTATTCCTTT
YZ*-SbTF*-R	TGGCACATCGAGCACA
*SbMNF*-F	ATGGCTATCTCCTCTCAAAACGACAAGCTA
*SbMNF*-R	TCATTCTCTAGATTCAACATATCCCGCTGC
YZ*-SbMNF*-F	CCCACTTCATCGCAGCTTGACTAACAGCTA
YZ*-SbMNF*-R	ATATTTTCTTCGGGTGGCACCATGCACACC

### 2.2. Phylogenetic tree construction

A phylogenetic analysis was conducted based on *SbTF* homologous proteins (Table 2). The sequence of each protein was independently aligned in MEGA6 (Tamura et al. 2013) using default parameters. Maximum likelihood analyses were conducted on the resulting concatenated dataset. The default model [Jones-Taylor-Thornton (JTT)] in MEGA was selected to establish the tree using the neighbour-joining method. The standard bootstrap method was selected to assess the phylogenetic support and the number of replicates was set to 500. Partial deletion was selected and the deletion degree was set to 50%. A consensus tree was then constructed, and clades with bootstrap support ≥50% were considered basically reliable (Hillis and Bull 1993).

**Table 2.** *SbTF* homologous proteins.

Description	Scientific name	Accession number
Malamycin response protein 1	*Paraphoma chrysanthemicola*	KAH7084744.1
Elsinochrome C biosynthesis regulatory protein elcr-like protein	*Parastagonospora nodorum*	KAH3966140.1
Aflatoxin biosynthesis regulatory protein	*Colletotrichum spinosum*	TDZ27476.1
Aflatoxin biosynthesis regulatory protein	*Cercospora beticola*	XP_023460061.1
Aflatoxin biosynthesis regulatory protein	*Elsinoe australis*	TKX25779.1
Transcriptional activator protein UGA3	*Elsinoe australis*	PSK53770.1
Transcriptional activator protein UGA3	*Sphaceloma murrayae*	PNS21564.1
Fungal Zn (2)-Cys (6) binuclear cluster domain-containing protein 2	*Elsinoe fawcettii*	KAF4548437.1
Cercosporin biosynthesis regulatory protein CTB8	*Fulvia fulva*	XP_047765394.1
Aflatoxin biosynthesis regulatory protein	*Colletotrichum orbiculare* MAFF 240422	TDZ26706.1
Monodictyphenone cluster transcription factor	*Colletotrichum trifolii*	TDZ40841.1
Putative aflatoxin regulatory protein	*Septoria linicola*	USW47939.1
Zn-II 2Cys6 regulatory protein	*Massariosphaeria phaeospora*	KAF2866751.1
Zinc finger transcription factor	*Colletotrichum graminicola* M1.001	XP_008097708.1
AFLR	*Aspergillus flavus*	AAM02994.1
C6 transcription factor (AFLR)	*Rasamsonia emersonii* CBS 393.64	XP_013324718.1
Transcription factor AFLR	*Aspergillus parasiticus*	AAD24767.1
Zinc finger transcription factor	*Shiraia* sp. slf14	AIW0Q663.1
Hypocrellin pathway transcription factor	*Shiraia* sp. SUPER-H168	AZP03050.1

### 2.3. Protoplast transformation of wild type strains

The wild type strains were cultured for 5 days at 26 °C in plates containing potato dextrose agar (PDA) media with yeast powder. To collect the spores, approximately 5 mL of 0.1% Tween 80 aqueous solution was poured onto the plate, and the spores were scraped from the plate with a toothpick. The mycelia were removed by straining through six layers of gauze. The spores were then obtained by centrifuging the filtrate and washed with 5 mL of sterile ddH_2_O. The washing and centrifugation were repeated twice. The spores were then resuspended in 30 mL potato dextrose broth (PDB) and cultured at 26 °C and 180 r/min until they germinated and grew three times in length. The precipitate was collected, and the germinating spores were washed with 5 mL sterile ddH_2_O twice. To prepare an enzymolysis solution, 2 mg of Yatalase and 3 mg of Lysozyme were added to 10 mL of OM buffer (Osmotics Medium) and sterilised by filtration. The germinating spores were added to the enzymolysis solution and digested at 37 °C and 80 r/min. Microscopic observation was performed every 30 min until most of the spores were converted into protoplasts. The protoplasts were preserved at −80 °C in 150 μL STC buffer. The abovementioned linearised plasmids were separately transformed into the protoplasts using a PEG-mediated method. The transformed protoplasts were incubated on a medium containing hygromycin B at 26 °C for 6–8 days, and single colonies were selected for subculture.

### 2.4. RNA isolation and quantitative real-time PCR (qRT-PCR)

The wild type and transformed strains were cultured in PDB without hygromycin B at 26 °C for 144 h. RNA was isolated from the strains using an M5 Plant RNeasy Mini Kit (Mei5 Biotechnology, Beijing, China). An M5 Sprint qPCR RT Kit with gDNA remover (Mei5 Biotechnology) was used to synthesise first-strand cDNA. qRT-PCR was performed using a Light Cycler 480II/96 (F. Hoffmann-La Roche, Basel, Switzerland).

### 2.5. High-performance liquid chromatography (HPLC)

The wild type and transformed strains were cultured in PDB, and then the mycelia were dried and soaked in methanol. The cell-associated hypocrellins were extracted as described previously for *S. bambusicola* (Shen et al. 2016). The content of the extracts was analysed using an Agilent 1200 Series HPLC system equipped with a Kromasil 100-5 C18 column (250 mm × 4.6 mm). Methanol (phase A) and 0.1% phosphoric acid in water (phase B) were used as the mobile phases, and gradient elution was performed according to the procedure in Table 3. The column temperature was 35 °C, the flow rate was 1 mL/min, and the loading volume was 20 μL. The signal was detected at 460 nm.

**Table 3.** HPLC gradient elution procedure for hypocrellin.

Time	Components
0–10 min	60% methanol, 40% 0.1% phosphoric acid
10–15 min	60%–70% methanol, 40%–30% 0.1% phosphoric acid
15–25 min	70% methanol, 30% 0.1% phosphoric acid
25–45 min	70%–75% methanol, 30%–25% 0.1% phosphoric acid
45–60 min	75%–100% methanol, 25%–0% 0.1% phosphoric acid
60–80 min	100% methanol

## 3. Results

### 3.1. Identification and characterisation of *SbTF* in *Shiraia*-like species

**Figure 1.**
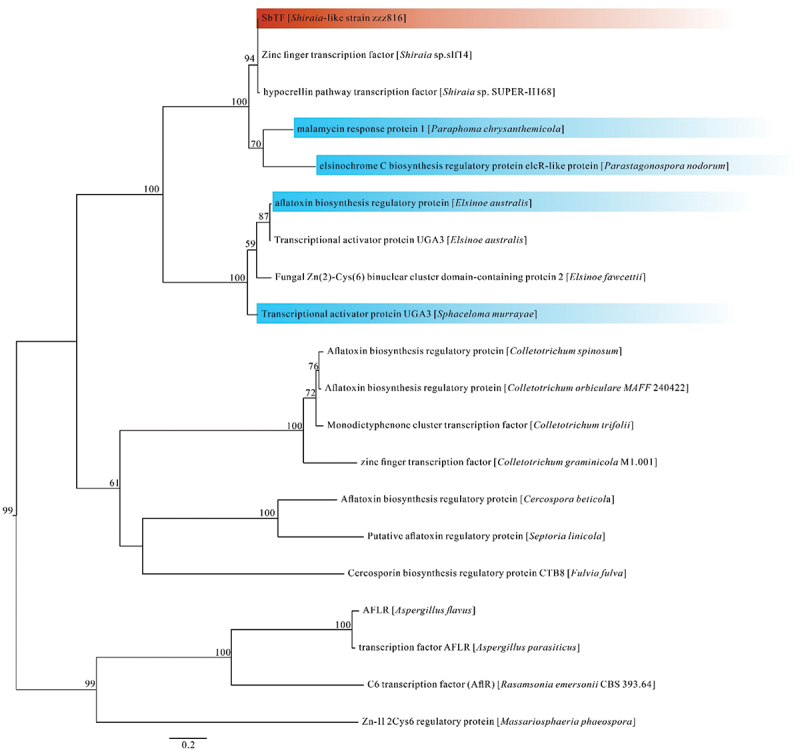
Phylogenetic tree of *SbTF* homologous proteins based on a maximum likelihood analysis of protein sequences. Maximum likelihood bootstrap support values (≥50%) are indicated above the nodes. The *Shiraia*-like strain zzz816 is shown in red, while other species with homologous transcription factors are shown in blue.

Based on comparative genomic and functional gene analyses, the *SbTF* gene was found in the hypocrellin biosynthesis gene cluster along with *SbPKS1* (*Shiraia bambusicola* polyketide synthase 1, which catalyses the formation of the PQ core). *SbPKS1* plays a crucial role in the synthesis of hypocrellins (a type of PQ produced by *Shiraia* and *Shiraia*-like species). The conserved AflR (aflatoxin pathway regulator) protein-coding region indicated that *SbTF* is a special transcription factor in this gene cluster. A phylogenetic tree of the *SbTF* homologous proteins was established using a maximum likelihood analysis (Figure 1).

In *Parastagonospora nodorum*, the homologous protein is the EC biosynthesis regulatory protein (Jones et al. 2021). In *Paraphoma chrysanthemicola*, the protein is known as the malamycin response protein 1 (Mesny et al. 2021). In *Elsinoe australis*, the protein has been described as the aflatoxin biosynthesis regulatory protein. In *Sphaceloma murrayae*, gene annotation suggests that the protein is the transcriptional activator protein UGA3. Based on these findings and the phylogenetic tree, it is speculated that the protein encoded by *SbTF* is involved in the regulation of hypocrellin synthesis, although some of the homologous proteins exhibit different regulatory functions.

**Figure 2.**
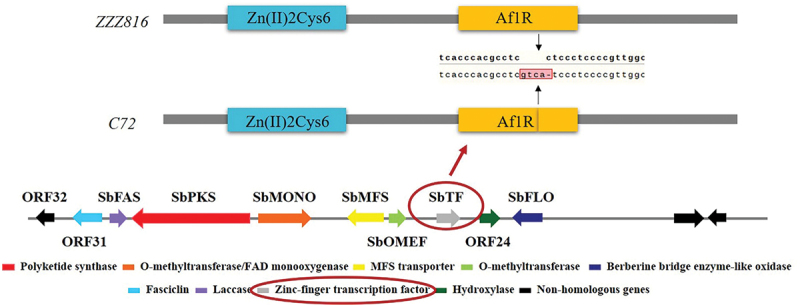
Variable region of *SbTF* gene between zzz816 and CNUCC C72.

Among the three hypocrellin-producing strains zzz816, CNUCC 1353PR, and CNUCC C72, there were distinct differences in phenotype under different culture conditions. Notably, under submerged fermentation, CNUCC 1353PR had a higher hypocrellin yield, zzz816 had a high and relatively stable hypocrellin yield, and CNUCC C72 had an almost undetectable hypocrellin yield. Based on comprehensive comparison of the *SbTF* sequence and *SbTF* expression in different strains, there was a frame shift mutation in *SbTF* CNUCC C72 compared to zzz816 (Figure 2) and this mutation seemed to impair the relevant functional domains, rendering the hypocrellin biosynthesis pathway inactive in CNUCC C72.

### 3.2. Analysis of *SbTF*-overexpressing transformants

**Figure 3.**
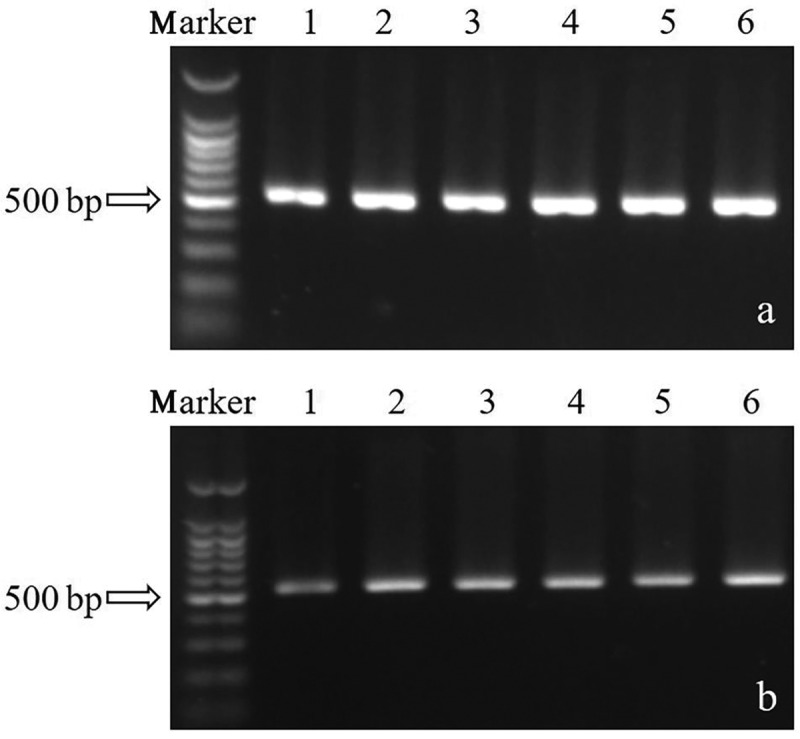
*SbTF* overexpression in (a) transformant zzz816 and (b) transformant CNUCC C72.

**Figure 4.**
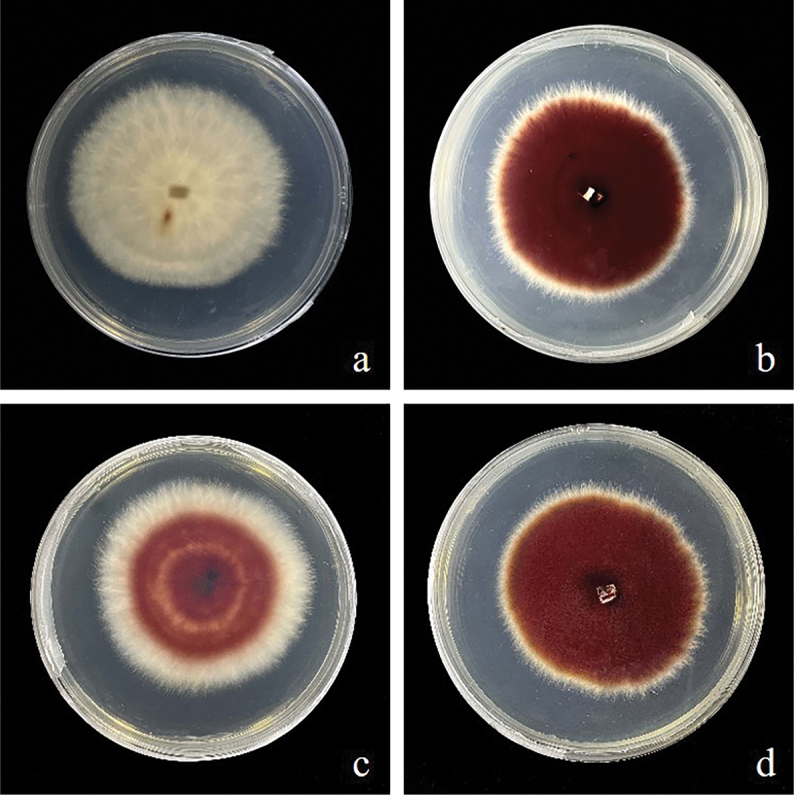
Images of wild type strains and *SbTF*-overexpressing transformants cultured for 144 h on PDA plates. (a) Wild type CNUCC C72. (b) Transformant CNUCC C72. (c) Wild type zzz816. (d) Transformant zzz816.

To improve hypocrellin production, the *SbTF* gene was cloned from the stable high-yielding strain zzz816 and transformed, under the control of the fungal promoter *PdgA*, into various strains [CNUCC 1353PR (high-yielding), zzz816 (high-yielding but unstable), and CNUCC C72 (low-yielding)] (Figure 3). *SbTF* overexpression had no significant effect on CNUCC 1353PR compared to the wild type (high-yielding strain). However, *SbTF* overexpression transformed the originally unstable zzz816 strain into a stable high-yielding strain, as shown by the HPLC results. *SbTF* overexpression significantly increased the hypocrellin production in CNUCC C72 compared to the wild type (low-yielding strain), resulting in a deep red colouration in culture (Figure 4).

**Figure 5.**
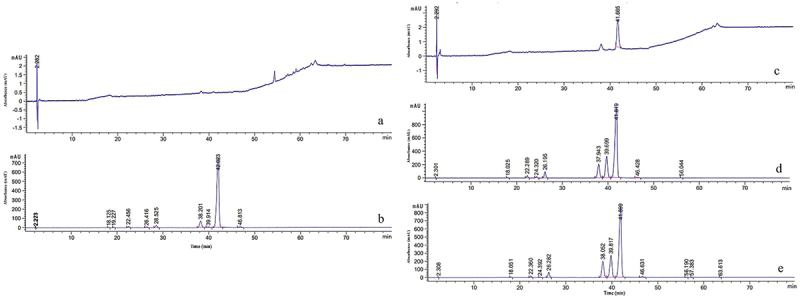
Comparison of wild type strains and *SbTF*-overexpressing transformants in liquid cultures. (a) Wild type CNUCC C72. (b) Transformant CNUCC C72. (c) Wild type zzz816 (low-yielding strain). (d) Wild type zzz816 (high-yielding strain). (e) Transformant zzz816.

HPLC analysis was employed to estimate the active components of final production. This showed that the hypocrellin yield was significantly higher in transformant zzz816 and transformant CNUCC C72 than the corresponding wild types (Figure 5). Using a standard curve for total hypocrellins, the HA, HB, and HC yield in transformant CNUCC C72 was 1,290, 40, and 237 mg/L, respectively, compared to no detection in the wild type. In transformant zzz816, the hypocrellin yield increased by 749.6 times that of the wild type. Notably, the hypocrellin yield of transformant zzz816 remained stable at a high level after five successive generations. *SbTF* overexpression improved the production and made the high-yielding strain more stable. In contrast, the transformant CNUCC 1353PR did not show such improvement, most likely due to the already extremely high yield of the wild type.

### 3.3. Changes in downstream gene expression

**Figure 6.**
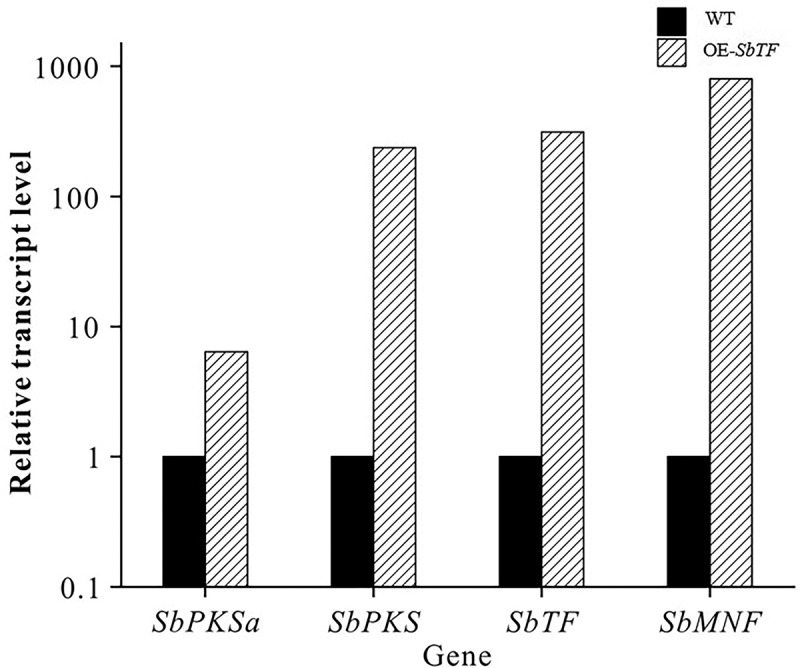
Comparison of qRT-PCR results between wild type and *SbTF*-overexpressing CNUCC C72.

**Figure 7.**
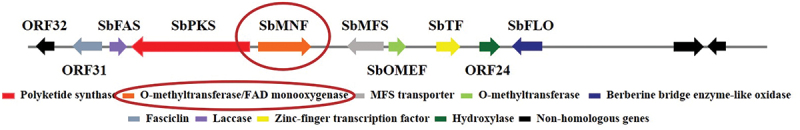
Location of *SbMNF* (putative monooxygenase gene) in hypocrellin biosynthesis gene cluster.

qRT-PCR was used to analyse the expression changes of relevant genes between wild type and *SbTF*-overexpressing CNUCC C72. *SbTF* expression increased by 312 times, and the key functional gene *SbPKS* increased by 237.5 times (Figure 6). Among the downstream genes, *SbMNF* showed the largest change in expression. *SbMNF* is an unknown functional gene in the hypocrellin biosynthesis gene cluster, but homologous genes have been annotated as monooxygenase genes based on conserved domains (Figure 7). Based on these findings, *SbMNF* overexpression experiments were conducted using various strains.

### 3.4. Effect of *SbMNF* overexpression on hypocrellin synthesis

**Figure 8.**
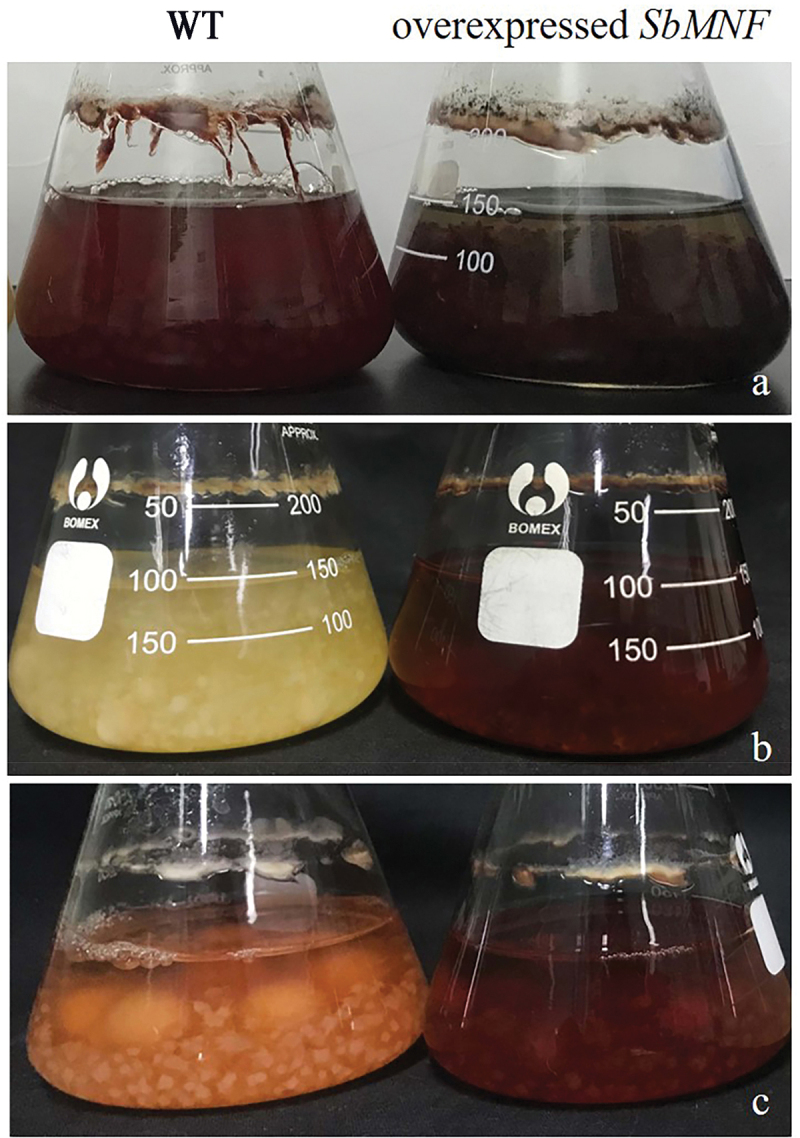
Images of wild type strains and *SbMNF*-overexpressing transformants cultured for 144 h in PDB. (a) Wild type and transformant CNUCC 1353PR. (b) Wild type and transformant CNUCC C72. (c) Wild type and transformant zzz816.

*SbMNF* overexpression in CNUCC 1353PR resulted in a darker black-red colour during the early stages of submerged fermentation, compared to the wild type (high-yielding strain). This significant increase in hypocrellin production occurred within the first 24 h of submerged fermentation, which was earlier than that in the wild type, which usually started synthesising hypocrellins after 48 h. This indicated a shorter fermentation cycle and improved production efficiency after genetic modification. The same change in phenotype was observed in CNUCC C72 and zzz816 (Figure 8).

**Figure 9.**
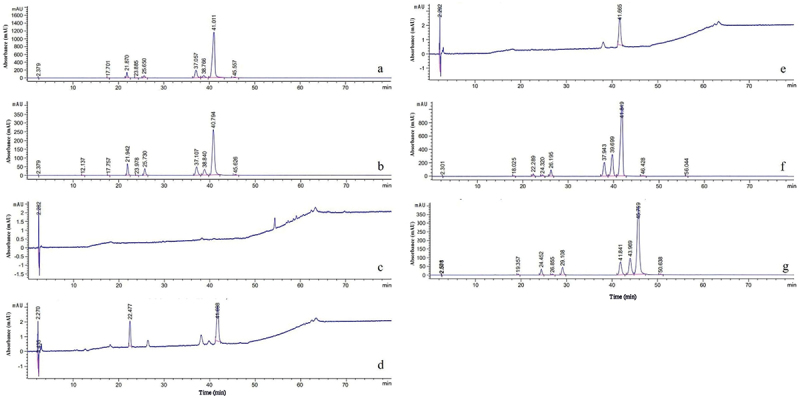
Comparison of wild type strains and *SbMNF*-overexpressing transformant in liquid cultures. (a) Wild type CNUCC 1353PR. (b) Transformant CNUCC 1353PR. (c) Wild type CNUCC C72. (d) Transformant CNUCC C72. (e) Wild type zzz816 (low-yielding strain). (f) Wild type zzz816 (high-yielding strain). (g) Transformant zzz816.

After 144 h of submerged fermentation, HPLC analysis showed significantly increased hypocrellin yields in the *SbMNF*-overexpressing transformants (Figure 9). In particular, the hypocrellin yield was 18 times higher in transformant CNUCC C72 than the wild type, according to the standard curve of total hypocrellins. The effects of *SbMNF* overexpression on CNUCC 1353PR and zzz816 differed. The EC yield was 2 times higher in CNUCC 1353PR than the wild type. In contrast, the HB yield was 39.4 and 70.3 times higher in zzz816 than the transformant and wild type, respectively. The HB yield increased by 7.03 times from 257 mg/L in the wild type to 1,807.5 mg/L in the transformant zzz816, almost reaching the HA yield in the wild type (1,873.7 mg/L), while the HA yield decreased by 14.01 times from 1,873.7 mg/L in the wild type to 133.7 mg/L in the transformant. The increase in EC in transformant CNUCC 1353PR was also accompanied by a significant decrease in HA.

## 4. Discussion

In this study, *SbTF* overexpression significantly increased the yield of target metabolites in CNUCC C72 (low-yielding strain) and restored stability to zzz816 (high but unstable yield). Improving high-yielding industrial strains may facilitate strain breeding, resulting in more stably high-yielding industrial strains for microbial fermentation. Additionally, analysis of downstream genes in the gene cluster revealed that *SbTF* overexpression had the largest impact on *SbMNF* expression. *SbMNF* is an unknown functional gene but homologous genes have been annotated as monooxygenase genes based on conserved protein domains. Building on this, we further assessed *SbMNF*- overexpressing strains.

In CNUCC 1353PR (high-yielding strain), *SbMNF* overexpression directly transformed hypocrellins into elsinochromes, another type of PQ. This finding represents the first alteration of the PQ framework during submerged fermentation of a transformant strain, where one active agent is converted into another. Furthermore, in zzz816, *SbMNF* overexpression led to the HB yield being significantly higher than the HA yield, which is typically considered the dominant component in most industrial strains. Therefore, *SbMNF* overexpression appears to produce different hypocrellins during submerged fermentation, resulting in improved production of rare compounds. Thus, although *SbMNF* is not the core enzyme in PQ synthesis, it can impact the modification of the chemical structure and even directly convert the original hypocrellins into different PQs. Our results demonstrate that *SbMNF* overexpression can increase the hypocrellin yield in a low-yielding strain and convert the active agent into different PQs during submerged fermentation. In the future, *SbMNF* is expected to play a larger role in the bioconversion of natural products and the breeding of low-yielding strains.

In this study, we analysed the functions of the transcription factor gene *SbTF* and the putative monooxygenase gene *SbMNF* in industrial strains with different properties. *SbTF* overexpression activated the hypocrellin biosynthesis pathway in the low-yielding strain, and stabilised the unstable high-yielding strain. Additionally, the non-essential gene *SbMNF* can assist in improving fermentation production. Moreover, *SbMNF* can convert native PQs into different types. This process could be further developed and applied in strain breeding to modify the composition of the final products.

The functional application of *SbTF* and *SbMNF* provides novel ideas for improving hypocrellin production and advancing the optimisation of relevant industrial strains.
